# Foodborne disease burden in Sweden with a One Health perspective

**DOI:** 10.1016/j.isci.2026.117023

**Published:** 2026-08-04

**Authors:** Ismael Halawa, Anna Omazic, Junwen Guo

**Affiliations:** 1 Department of Epidemiology and Global Health, Umeå University, 901 85 Umeå, Sweden; 2 Department of Chemistry, Environment and Feed Hygiene, Swedish Veterinary Agency (SVA), 751 89 Uppsala, Sweden

**Keywords:** One Health, foodborne disease, climate change, food safety, DALY

## Abstract

Foodborne diseases (FBDs) pose a substantial public health burden worldwide, yet their extent and drivers in Sweden have not been comprehensively assessed. Using a One Health framework, we estimated the national FBD burden from 2013 to 2022 and explored links to zoonotic sources and climate factors. We identified over 1.2 million cases, most unreported, corresponding to 37,665 disability-adjusted life years (DALYs). Campylobacteriosis accounted for the largest share of cases and disease burden, with strong correlations to poultry farm outbreaks and contaminated chicken meat, while salmonellosis ranked second in disease burden. Listeriosis caused relatively few cases, yet a disproportionately high disease burden. Increased temperature and precipitation were associated with higher annual incidence. Higher population-adjusted burden was observed in northern counties. These findings provide a deep understanding of FBD dynamics in Sweden and support the need for strengthened prevention and surveillance strategies across the food production chain.

## Highlights

• From 2013 to 2022, foodborne diseases caused 1.2 million cases and 37,665 DALYs in Sweden
• Campylobacteriosis and salmonellosis have the highest DALY burden among others
• Strong links between human campylobacteriosis cases and poultry chicken farm outbreaks
• Associations are observed between bioclimatic variables and annual foodborne disease cases

## Graphical abstract

**Figure:**
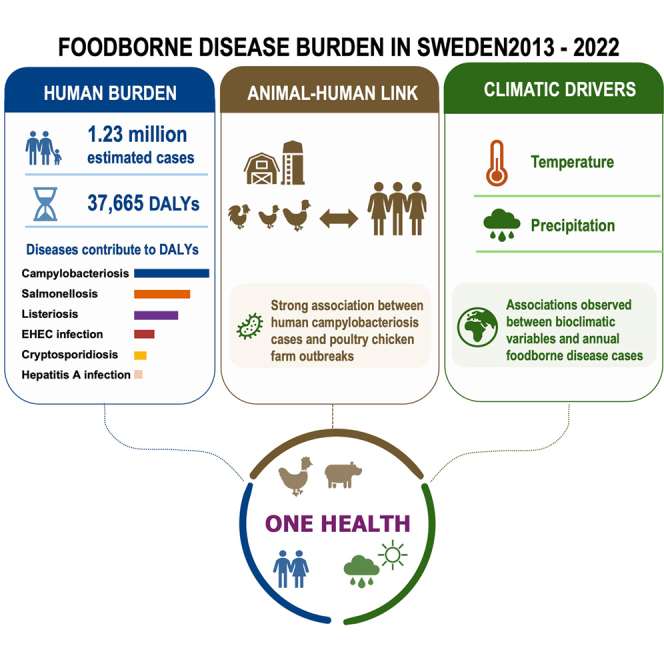

## Teaser

Public health; Food science; Food safety; Foodborne illness

## Introduction

Foodborne diseases (FBDs) have been a major global public health issue for decades. In 2010, over 600 million people across the world fell ill and 420,000 died due to FBDs according to estimates from the World Health Organization (WHO), equating to a loss of 33 million disability-adjusted life years (DALYs).^1^ FBDs are often associated with the consumption of contaminated food or beverages. There are over 200 known pathogens associated with FBDs. These include bacteria, parasites, viruses, chemicals, and toxins that can cause a wide range of symptoms.^2^

In 2023, approximately 300,000 confirmed cases of human FBDs were reported in Europe to the European Centre for Disease Prevention and Control (ECDC). This included roughly 10,000 cases from Sweden, costing the national healthcare sector an estimated €142 million annually.^3^ However, the actual number of cases may be significantly higher than the reported figures, due to underreporting and insufficient surveillance capacity in practice.^1^ A recent Danish study estimated that the actual FBD cases could be 10 to 20 times higher than those officially reported,^4^ a trend also observed in another European study.^5^ Moreover, environmental and climatic changes are expected to increase the incidence of FBDs by introducing new transmission routes and intensifying infections caused by pathogens, such as *Campylobacter* and *Salmonella*.^6^^,^^7^

Most FBDs are strongly influenced by multiple factors, such as climate change, food safety, and animal health, paving the way for novel transmission pathways for FBDs.^8^ Previous studies have shown that climate-related factors, such as temperature and humidity, can have direct effects on FBDs by influencing the growth, survival, and persistence of pathogens in food.^9^ For example, higher ambient temperatures can accelerate bacterial replication, while excessive moisture can facilitate pathogen survival on surfaces and in foods. In addition to these direct biological effects, climate variables can also affect indirect pathways, including food production environments, hygiene practices, storage and transport conditions, and overall contamination risk along the food chain.^10^ Therefore, applying a One Health approach that integrates animal, environmental, and human health is essential for effectively studying and addressing FBDs. To our knowledge, no study has quantified the FBD burden specifically in Sweden nor applied a One Health perspective.

In this study, we aim to explore six common FBDs in Sweden: campylobacteriosis, cryptosporidiosis, enterohaemorrhagic E. coli (EHEC) infection, hepatitis A infection, listeriosis, and non-typhoidal salmonellosis. This selection reflects the pathogens for which Sweden maintains consistent, high-quality national surveillance data suitable for DALY estimation. Several relevant pathogens, such as *norovirus*, could not be included because they were not notifiable during the study period. Therefore, the analysis focuses on the aforementioned pathogens for which robust national data exist, ensuring comparability and validity. This study first quantifies the DALY burden associated with FBDs in Sweden and then applies a One Health perspective to understand the complex interactions among humans, animals, and the environment. Using the available data, we aim to examine linkages between zoonotic and human FBD incidence and to identify how bioclimatic factors influence the incidence of FBDs.

## Results

### FBDs in Sweden

**Figure 1:**
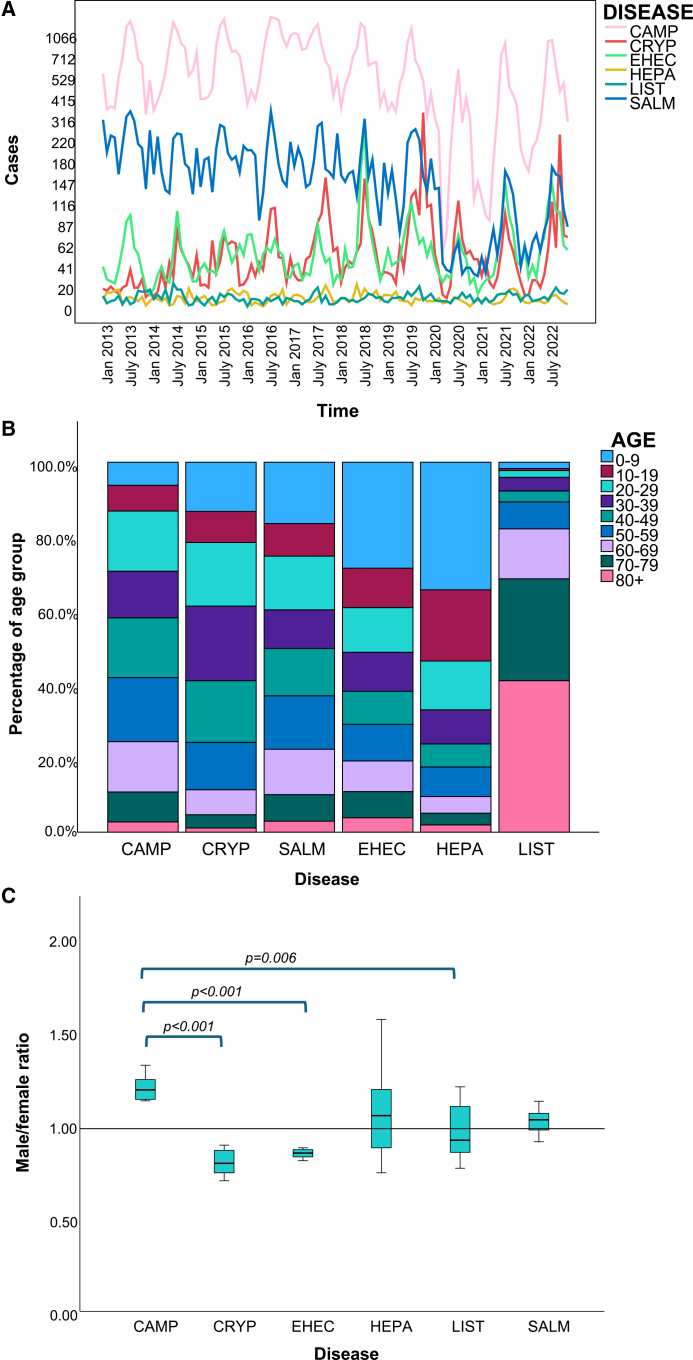
The overview of six foodborne diseases in Sweden from 2013 to 2022 (A) Monthly incidence trend, (B) age distribution, and (C) the male/female ratio of each disease. The significant male/female ratio across groups is visualized in the boxplot. Statistical significance was defined as *p* < 0.05. CAMP, campylobacteriosis; CRYP, cryptosporidiosis; SALM, salmonellosis; EHEC, enterohemorrhagic E. coli infection; HEPA, hepatitis A infection; and LIST, listeriosis.

Between 2013 and 2022, the distribution of FBDs fluctuated. Still, the overall trends remained stable (Figure 1A). There was a notable drop in reported cases of FBDs from 2020 onwards, coinciding with the onset of the COVID-19 pandemic. The monthly temporal pattern of reported FBDs indicated that campylobacteriosis consistently accounted for the highest number of reported FBD cases, with prominent annual spikes observed during the summer months, as seen also with salmonellosis, cryptosporidiosis, and EHEC infection. Hepatitis A infection and listeriosis had low and stable case numbers throughout the period, with minimal seasonal fluctuations. Most FBDs displayed summer incidence peaks, but no clear long-term temporal trend was observed (Figure 1A).

Most FBDs (campylobacteriosis, cryptosporidiosis, salmonellosis, EHEC infection, and hepatitis A infection) were evenly distributed across all age groups, with no strong age-specific trends, except an increasing incidence trend in the 0–9 age group in the respective order (Figure 1B). On the other hand, listeriosis showed a distinct pattern, disproportionately affecting older adults, particularly the 80+ age group, with the cumulative age groups 0–59 making up less than 20% of the total incidence (Figure 1B). The male/female ratio distribution of the FBDs in Sweden revealed three distinct gender disparities (Figure 1C). Both cryptosporidiosis and EHEC infection showed a consistent female predominance with gender ratios of 0.8 and 0.9, respectively. These findings were significantly different from the male predominance of campylobacteriosis, which had a gender ratio of 1.1 (Kruskal-Wallis test, *p* < 0.05). In contrast, salmonellosis, listeriosis, and hepatitis A infection exhibited a balanced distribution between genders without any gender predominance in the interquartile range.

### Estimated disease incidence and DALY

The reported incidence in Sweden across the six FDBs studied between 2013 and 2022 was 107,972 cases, whereas the estimated cases exceeded 2 million, with approximately 1.2 million cases suspected to be foodborne. Overall, FBDs accounted for 37,665 DALYs, as shown in Table 1. The FBD with the highest incidence and disease burden was campylobacteriosis, where the actual foodborne cases were estimated to be close to 1 million, in comparison with the reported incidence of just under 75,000 cases. Its disease burden amounted to over half of the total FBD burden, at 19,415 DALYs. The FBD with the lowest estimated FBD burden was hepatitis A infection, which was estimated to be 173 DALYs and approximately 1,700 cases. Listeriosis was estimated to have the lowest incidence at under 1,000 cases. However, strongly amplified by its high per-case DALY, it ranked third in overall FBD burden (Table 1).

**Table 1:** Estimations of foodborne diseases burden in Sweden

Disease	Reported cases^11^	Underreporting multiplier^12^^,^^13^^,^^14^	DALY per case^15^	FBD attribution^11^	Estimated cases	Estimated FBD cases	Estimated FBD DALY
Campylobacteriosis	74,695	17.1	0.02	76%	1,277,285	970,737	19,415
Salmonellosis	18,809	10.2	0.06	76%	191,852	145,808	8,748
Listeriosis	977	1	8	100%	977	977	7,816
EHEC infection	6,365	13.3	0.02	60%	84,655	50,793	1,016
Cryptosporidiosis	6,212	100	0.008	10%	621,200	62,120	497
Hepatitis A infection	914	4.5	0.1	42%	4,113	1,727	173
**Total**	**107,972**				**2,180,082**	**1,232,162**	**37,665**

Estimated foodborne incidence and disease burden measured in disability-adjusted life year (DALY) in Sweden for six FBDs. Reported cases reflect yearly cumulative cases reported from 2013 to 2022. Bold values indicate the total estimated cases/burdens across all included foodborne diseases.

**Figure 2:**
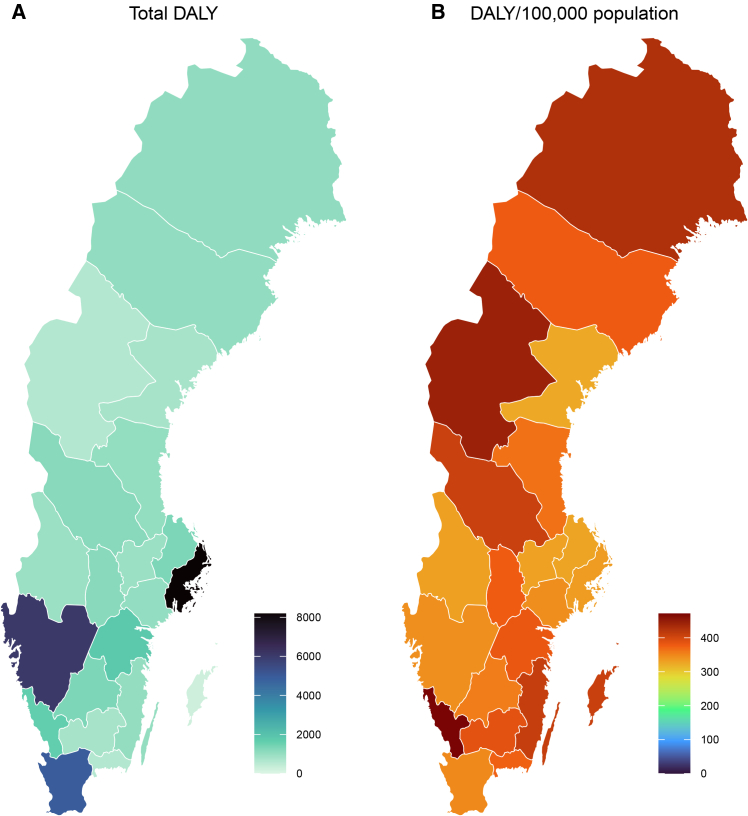
County distribution of disability-adjusted life years for six foodborne diseases in Sweden (2013–2022) The map visualizes (A) the total reported disability-adjusted life years (DALY) and (B) the total reported DALY adjusted per 100,000 people across Sweden’s 21 counties.

The FBD burden varied across the counties (Figure 2). Although most cases were reported in the three most populated counties of Sweden (Stockholm, Skåne, and Västra Götaland) (Figure 2A), when adjusted to population, it was more evident that other counties were more greatly affected by the burden, particularly some northern counties (Figure 2B). The counties with the highest population-adjusted DALYs were Halland, Jämtland, and Norrbotten.

### Primary food sources associated with FBDs

**Figure 3:**
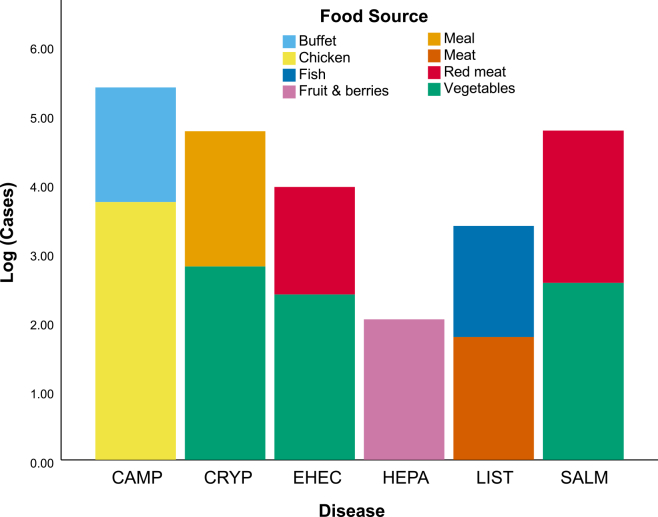
Foodborne diseases and their suspected food sources in Sweden based on the traceable outbreak investigation data from the Swedish Food Agency Bars show the most frequently reported food vehicles among suspected and confirmed outbreak investigations notified to the Swedish Food Agency from 2013 to 2022; only events with a traceable food source are included, and the categories “Unknown” and “Other” are excluded.

During 2013 and 2022, based on the data from traceable outbreak investigations, FBDs were linked to various food types. Among the pathogens studied, *Campylobacter* accounted for the majority of reported cases where the food source was identified, with chicken meat being the most frequently reported food source. Vegetable consumption was most often attributed to EHEC infection, cryptosporidiosis, and salmonellosis. Listeriosis was associated with meat consumption. Lastly, hepatitis A infection was most often linked to fruit and berries (Figure 3). Overall, chicken meat and vegetables were the primary sources of all traceable Swedish FBD cases.

### Regional overview

**Figure 4:**
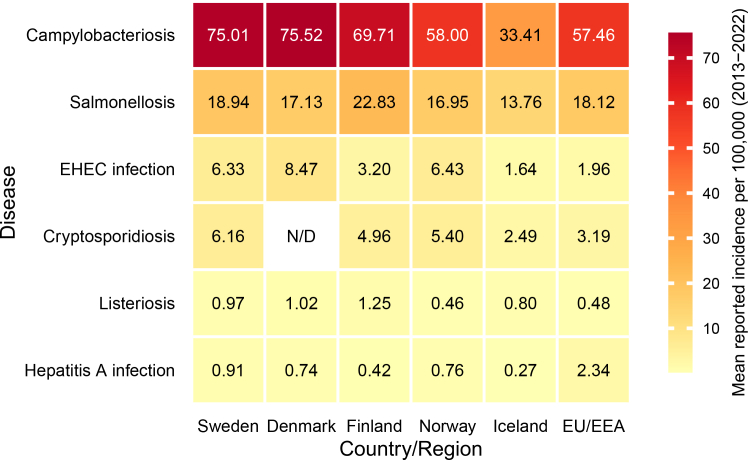
Regional foodborne diseases overview The heatmap shows the mean annual reported incidence per 100,000 population for six notifiable enteric diseases campylobacteriosis, salmonellosis, EHEC infection, cryptosporidiosis, listeriosis, and hepatitis A infection, across Sweden, Denmark, Finland, Norway, Iceland, and the EU/EEA, over 2013–2022. Differences in surveillance intensity, diagnostics, case definitions, and outbreak activity may influence reported rates.

Compared to the incidence rate of FBDs in neighboring Nordic countries and the EU/EEA (Figure 4), campylobacteriosis had the highest mean incidence across the Nordics (peaking in Denmark and Sweden ∼75/100,000 population) and exceeded the EU/EEA average in all but Iceland. Salmonellosis and cryptosporidiosis showed intermediate levels, with Finland having the highest incidence of salmonellosis level and Denmark lacking cryptosporidiosis data. EHEC infection was notably higher in Denmark and Norway. Listeriosis and hepatitis A infection remained low throughout the Nordics, with hepatitis A infection well below the EU/EEA average. However, when interpreting these geographic patterns, it is important to note that differences in surveillance intensity, diagnostics, case definitions, and localized outbreak activity may influence the reported rates across counties.

### One Health investigation of FBDs

#### Human-chicken correlation with campylobacteriosis

**Figure 5:**
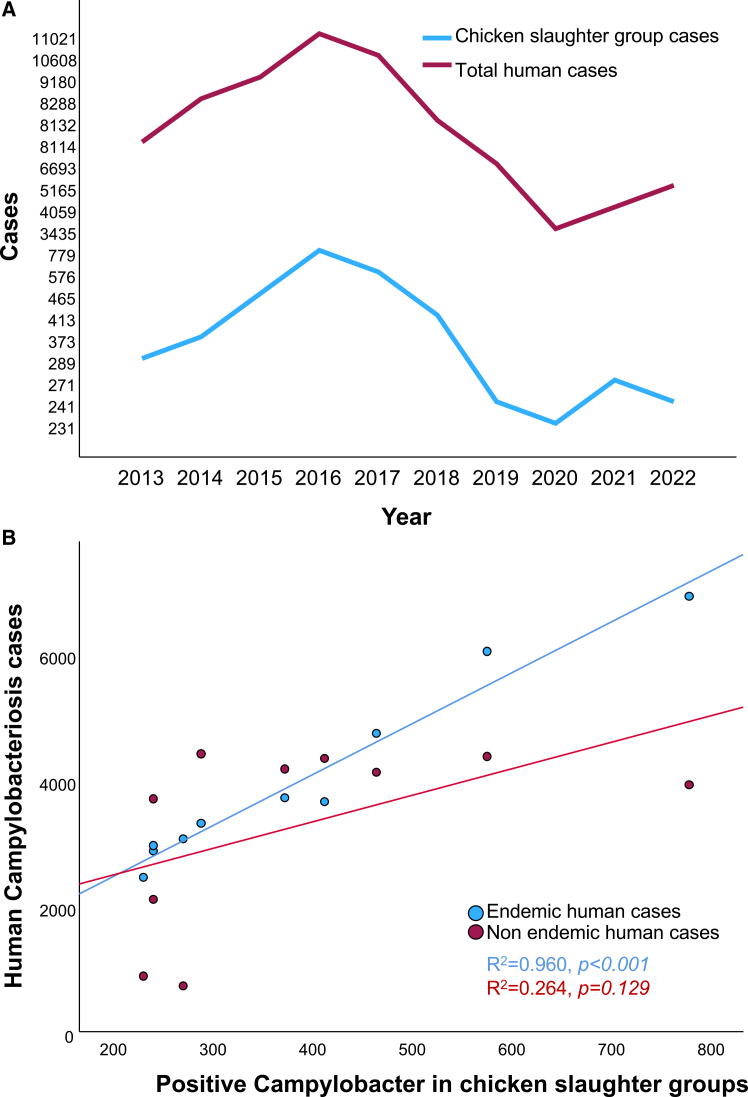
Slaughter chicken and human incidence analysis The line graph (A) illustrates the annual number of reported endemic cases of campylobacteriosis in humans (in red) and positive *Campylobacter* tests in broiler chicken slaughter groups (in blue) in Sweden from 2013 to 2022. Positive tests in chickens were based on duodenal sampling, where a group is considered positive if at least 10 samples test positive. The scatterplot (B) compares reported human cases of campylobacteriosis (on the *y* axis) with positive *Campylobacter* tests in chicken slaughter groups (on the *x* axis). It separates endemic human cases (disease occurring in Sweden) using blue points from non-endemic cases (disease occurring abroad but reported in Sweden) using red points. The lines represent linear regressions for each group with associated *R*^*2*^ and *p* values. Statistical significance was defined as *p* < 0.05.

In Sweden, there were similar trends over the study period between the total reported cases of human campylobacteriosis and the positive cases found in chicken slaughter groups (Figure 5A). When the human endemic and non-endemic cases were separated, a significant correlation was discovered between endemic human cases and the carriership of *Campylobacter* in chickens (*R*^*2*^ = 0.960, *p* < 0.001) (Figure 5B). Conversely, there was a weaker, non-significant correlation for non-endemic cases (*R*^*2*^ = 0.264, *p* < 0.129).

### Climatic impact on FBDs

Our analysis identified several significant associations between bioclimatic variables and FBD incidence in Sweden (Table 2; Tables S1–S6). Precipitation-related variables, especially annual precipitation and precipitation during the warmest and driest quarters, were more strongly associated with FBD cases than temperature-related factors. Across all six pathogens, no significant temporal trend was observed, as the “Year” variable was not significant in any model. In contrast, the county was a significant factor for all pathogens, indicating notable spatial variation in incidence across Sweden.

**Table 2:** Results of the generalized linear model analysis examining the relationship between all Swedish reported cases of six foodborne diseases and bioclimatic variables using annual data from 2013 to 2022

Model	Salmonellosis			Campylobacteriosis			Listeriosis			Hepatitis A infection			Hepatitis A infection			Cryptosporidiosis		
GLM: negative binomial log	Wald chi-square	df	*p*	Wald chi-square	df	*p*	Wald chi-square	df	*p*	Wald chi-square	df	*p*	Wald chi-square	df	*p*	Wald chi-square	df	*p*
(Intercept)	0.664	1	0.415	0.141	1	0.707	0.011	1	0.915	0.266	1	0.606	2.695	1	0.101	0.61	1	0.435
Year	4.055	9	0.908	6.603	9	0.678	6.29	9	0.711	5.575	9	0.782	6.091	9	0.731	6.616	9	0.677
County	100.323	20	**<0.001**	96.812	20	**<0.001**	35.37	20	**0.018**	34.141	20	**0.025**	51.395	20	**<0.001**	79.057	20	**<0.001**
Annual mean temperature (Bio1)	0.316	1	0.574	0.521	1	0.47	0.355	1	0.551	0.072	1	0.789	0.295	1	0.587	1.266	1	0.261
Isothermality (Bio3)	2.182	1	0.14	2.799	1	0.094	0.539	1	0.463	0.022	1	0.882	3.381	1	0.066	0.609	1	0.435
Temperature seasonality (Bio4)	0.163	1	0.686	0.764	1	0.382	2.442	1	0.118	0.483	1	0.487	1.127	1	0.288	3.797	1	0.051
Max temperature of warmest month (Bio5)	0.863	1	0.353	0.52	1	0.471	0.483	1	0.487	0.085	1	0.77	0.266	1	0.606	1.098	1	0.295
Mean temperature of wettest quarter (Bio8)	2.629	1	0.105	4.161	1	**0.041**	2.9	1	0.089	0.823	1	0.364	0.511	1	0.475	6.624	1	**0.01**
Mean temperature of driest quarter (Bio9)	0.208	1	0.649	0	1	0.995	0.731	1	0.392	0.115	1	0.734	0.357	1	0.55	1.003	1	0.317
Annual precipitation (Bio12)	4.424	1	**0.035**	6.642	1	**0.01**	5.782	1	**0.016**	2.493	1	0.114	2.192	1	0.139	3.379	1	0.066
Precipitation of driest month (Bio14)	0.733	1	0.392	1.004	1	0.316	2.226	1	0.136	1.04	1	0.308	0.349	1	0.555	0.368	1	0.544
Precipitation seasonality (Bio15)	0.034	1	0.853	0.092	1	0.762	0.938	1	0.333	0.008	1	0.931	1.004	1	0.316	4.304	1	0.038
Precipitation of driest quarter (Bio17)	0.415	1	0.519	2.203	1	0.138	4.544	1	**0.033**	0.951	1	0.329	1.463	1	0.226	8.717	1	**0.003**
Precipitation of warmest quarter (Bio18)	6.826	1	**0.009**	8.475	1	**0.004**	12.444	1	**<0.001**	1.363	1	0.243	0.092	1	0.761	3.373	1	0.066
Precipitation of coldest quarter (Bio19)	0	1	0.999	0.535	1	0.464	1.683	1	0.195	4.282	1	**0.039**	1.275	1	0.259	2.123	1	0.145

For salmonellosis, we observed significant associations with annual precipitation (*p* = 0.040) and precipitation during the warmest quarter (*p* = 0.010). For campylobacteriosis, model estimates indicated that mean temperature of the wettest quarter (*p* = 0.041), annual precipitation (*p* = 0.010), and precipitation of the warmest quarter (*p* = 0.004) were significantly linked. Listeriosis incidence was associated with annual precipitation (*p* = 0.016), precipitation during the driest quarter (*p* = 0.033), and particularly with precipitation during the warmest quarter (*p* < 0.001).

For hepatitis A, a significant association was found with precipitation during the coldest quarter (*p* = 0.040). In the case of EHEC infection, no bioclimatic variables were statistically significant. cryptosporidiosis was significantly associated with the mean temperature of the wettest quarter (*p* = 0.010), precipitation seasonality (*p* = 0.038), and precipitation during the driest quarter (*p* = 0.003).

## Discussion

This study shows that campylobacteriosis, salmonellosis, and listeriosis were the primary contributors of FBD burden in Sweden. Listeriosis stands out for having a disproportionately high impact despite its relatively low incidence rates. We found evidence that over 1 million cases of FBDs were underreported during the study period. Geographically, some counties in northern Sweden showed higher FBD burden when incidence rates were adjusted for county population. These differences could be partly shaped by climate-related factors, such as seasonal changes in temperature and rainfall, which partially direct or indirectly influence the occurrence of FBDs. Additionally, we found the broiler chicken *Campylobacter* carriership in Swedish poultry farms were closely linked to endemically acquired human campylobacteriosis, highlighting the strong link between contaminated chicken meat as the primary source of campylobacteriosis. These findings underscore the necessity of an integrated One Health approach in managing FBD risks across animal, environmental, and human contexts in Sweden. Overall, the incidence of reported FBDs in Sweden is broadly comparable to that observed in other Nordic countries but notable differences include relatively higher incidences of campylobacteriosis, EHEC infection, and cryptoporidiosis in Sweden compared with several Nordic countries and the EU/EEA mean. However, these observed differences may also partly reflect heterogeneity in surveillance systems and clinical diagnostic practices across EU/EEA countries and should therefore be interpreted with caution.

The annual FBD burden in Sweden is comparable to the combined annual disease burden of HIV/AIDS and Tuberculosis in Sweden^16^ and about 5% of the disease burden of COVID-19 in Sweden from 2020 to 2021.^17^ Looking at the county distribution of reported cases in Sweden, most cases, and, subsequently, the highest disease burden was associated with counties with the highest populations. However, after adjusting for population differences, a markedly different distribution emerged, with the reported disease burden highest in the northern counties, except for one county on Sweden’s southwest coast (Halland). Local reporting practices, variations in animal management, local food consumption patterns, and climatic factors may be contributing to these county differences.^2^ The variation may also be influenced by local laboratory practices, healthcare availability, financial constraints in healthcare sectors, and other political factors, all of which have been observed in previous studies that influence the reporting of notifiable diseases.^18^^,^^19^

A significant decrease in reported cases from 2020 to 2021, concurrent with the COVID-19 pandemic and the following post-pandemic period, was observed for most FBDs. There could be many different reasons for this. One reason is, possibly, the decrease of patients seeking healthcare for FBDs.^20^ Furthermore, limited healthcare resources, particularly for infectious diseases, due to the heavily constrained healthcare systems during the pandemic could have significantly impacted the reported incidence of FBDs.^21^ Numerous studies have shown that many non-COVID-19 diseases reportedly decreased substantially during the pandemic.^20^^,^^22^ Furthermore, improved hygiene practices^23^ and reduced restaurant visits likely contributed to this decline.^24^ It is important to note that our generalized linear model analyses included the full study period, including pandemic years (2020–2022), which likely introduced noise into the model’s temporal component. Given that FBDs have high baseline noise from non-climatic factors such as travel, retaining the full dataset is essential for robust long-term bioclimatic evaluation. Future modeling exploring the pandemic’s impact alongside endemic cases could benefit from better isolation of true climatic drivers from acute pandemic-related behavioral disruptions.

The burden of FBDs affects the population differently across gender and age groups. Our study shows that there are significant differences in the gender distribution among various Swedish FBDs. Previous literature has noted the male predominance in campylobacteriosis, but the underlying reason remains unknown.^25^ The female predominance for cryptosporidiosis and EHEC infection is also mostly unknown. However, since women often consume more vegetables than men do,^26^ they may face a higher risk of exposure, as vegetables were the most reported food source in *Cryptosporidium* outbreaks according to our findings. Of the six FBDs studied, only listeriosis showed a marked age difference: most people falling ill had reached the age of 80. People in this age group and other immunosuppressed populations are particularly vulnerable to FBDs. The disproportionate age distribution for listeriosis is unknown, but, given its rarity, it is suspected that there are low levels of population immunity; thus, if the disease is contracted in old age, the weakened immune system struggles to fight the infection. In this study, we applied an underreporting multiplier of 1 for listeriosis, assuming complete case capture. While this is a standard assumption in similar European studies, it is important to acknowledge the sensitivity of this estimate. Given that listeriosis already accounts for the third-highest DALY burden even under this conservative assumption, any actual underreporting in practice would mean its true public health impact is even more severe.

### One Health perspective on FBDs

FBDs should not be considered solely within human health issue. Instead, they involve the complex interplay of humans, animals, and the environment, necessitating a One Health approach to identify and understand their contributing factors.^8^^,^^27^ A Danish study revealed that higher humidity and temperatures before chicken slaughter and the maximum temperature experienced by humans before infection were associated with increased infection rates.^28^ Consistent with these findings, our study showed a strong linkage between human endemic infection and *Campylobacter* carriership in broiler chickens. In addition to the national *Campylobacter* program, Sweden has a similar national *Salmonella* program. However, salmonellosis is occasionally detected in feed through targeted testing, both at production sites and on farms during disease investigations, this type of surveillance lacks the systematic structure and continuity. For this reason, we ultimately excluded the *Salmonella* program from our analysis. Nevertheless, additional effects must be considered in future systematic animal monitoring of FBD-related pathogens. Moreover, animal infection surveillance programs primarily target livestock meat production chains, while vegetables and fruit, significant sources of FBD burden, are not routinely subject to pathogen surveillance programs in Sweden. Instead, vegetable surveillance is monitored by multiple agencies. For instance, the Board of Agriculture oversees plant protection, county administrations inspect growers, municipalities regulate retailers and packing facilities and the Swedish Food Agency investigates outbreaks. Imported vegetables generally circulate freely within the EU, with only high-risk products subject to border checks. Some food items from specific countries are banned.

We found that increases in annual precipitation and precipitation during the warmest quarter were significantly associated with higher incidence rates of the three most burdensome FBDs studied: salmonellosis, campylobacteriosis, and listeriosis. Overall, precipitation-related variables emerged as key drivers of disease incidence across all FBDs. Previous studies have similarly demonstrated associations between climatic variables and FBDs, particularly precipitation and temperature, which are known to affect the persistence and spread of bacterial foodborne pathogens.^7^^,^^28^ These findings support the notion that future climate changes, involving increased rainfall and rising temperatures, may synergistically contribute to a higher burden of FBDs. However, we cannot draw definitive conclusions at the county level, and it is important to emphasize that the climatic variables used in this study represent large-scale change indicators rather than mechanistic exposure metrics. Therefore, associations should be viewed as exploratory patterns rather than proof of direct causal relationships, because many indirect pathways between climate and FBD outbreaks exist (e.g., effects on storage, handling, or food-processing environments). Mechanistic factors such as food transport, refrigeration failure, or processing-environment contamination were outside the scope of this study and require targeted data from microbiological and food-chain investigations, which are often lacking.

In conclusion, this study provides a clear look at the 10-year distribution of six FBDs in Sweden. Campylobacteriosis, salmonellosis, and listeriosis are the dominant contributors to the FBD burden in Sweden. Although listeriosis occurs at low incidence, it accounts for a disproportionately high disease burden, highlighting its public health relevance. Spatially, the total DALY burden was highest in southern Sweden, while several northern counties exhibited higher population-adjusted burdens, highlighting substantial regional heterogeneity. These geographic patterns may be partly shaped by climatic factors, such as changes in temperature and precipitation, which were associated with FBD incidence in our analyses. In addition, the strong association between *Campylobacter* carriage in broiler chickens and endemically acquired human campylobacteriosis reinforces the role of contaminated poultry meat as a key transmission pathway in Sweden.

Overall, these findings emphasize the importance of an integrated One Health perspective that jointly considers human health, animal, and environmental drivers when assessing and managing FBD risks. In light of our findings, near-term priorities should focus on (1) strengthening control along the poultry chain, from on-farm biosecurity through slaughter-hygiene and retail handling, to curb endemic campylobacteriosis; (2) incorporating climate-aware risk management (e.g., heightened monitoring and hygiene controls during warm, wet periods); and (3) strengthening surveillance for non-livestock foods, especially fresh vegetables (e.g., leafy greens, salad mixes, herbs) and imported produce/berries, by integrating currently fragmented data streams across agencies. Outbreak source data should continue to be used as signals of traceable vehicles rather than population-level exposure fractions, and counties with higher population-adjusted burdens may benefit from tailored monitoring and prevention strategies that reflect local food systems and testing capacity. Looking ahead, shared One Health data platforms that routinely link human, animal, food, and climate data, coupled with targeted microbiological and food-chain investigations, will enable more rigorous sensitivity testing and clearer causal insight; guiding more precise, preventive food-safety actions in Sweden.

### Limitations of the study

This study has several limitations. First, estimating the DALYs relies on underreporting multipliers (UM). Although we reviewed the available literature, especially the Nordic data, some parameters related to underreporting are not available for Sweden. Therefore, the true disease burden could be skewed. Second, our attribution of food sources is based on traceable outbreak data instead of population-level exposure fractions, since the capacity of individual investment remains very limited. Third, the spatial and temporal heterogeneity observed may be partially influenced by regional differences in surveillance intensity, diagnostics, and reporting practices. Finally, while our models identified significant bioclimatic associations, these variables serve as large-scale climatic indicators. The lack of high-resolution data to completely isolate travel-related imported cases from local infections at the regional level inherently introduced noise into the spatial-temporal analyses.

## Resource availability

### Lead contact

Requests for further information and resources should be directed to and will be fulfilled by the lead contact, Junwen Guo (junwen.guo@umu.se).

### Materials availability

This study did not generate any new materials.

### Data and code availability

• Data: all data analyzed in this study are publicly available from the Public Health Agency of Sweden (https://www.folkhalsomyndigheten.se/statistik-och-data/hitta-statistik-och-data/). Additional outbreak investigation data were obtained from the Swedish Food Agency and poultry surveillance data from the Swedish Veterinary Agency as described in the STAR Methods.
• Code: statistical analyses were performed using IBM SPSS Statistics version 29. No custom code was generated in this study.
• Other items: any additional information required to reanalyze the data reported in this paper is available from the lead contact upon request.

## Acknowledgments

The authors would like to sincerely thank Nabil Yousef, Chief Government Inspector at the Swedish Food Agency, for providing invaluable data on foodborne outbreaks in Sweden and answering important questions regarding the structure of the Swedish and European food safety system.

## Author contributions

I.H., data curation, investigation, formal analysis, visualization, and writing – original draft; A.O., conceptualization, supervision, validation, writing – review and editing; J.G., conceptualization, data curation, formal analysis, investigation, methodology, software, supervision, validation, and writing – review and editing.

## Declaration of interests

All authors declare that they have no conflicts of interest to disclose.

## STAR★Methods

### Key resources table

**Table:** 

REAGENT or RESOURCE	SOURCE	IDENTIFIER
**Deposited data**		

Bioclimatic variables	Copernicus Climate Data Store	https://cds.climate.copernicus.eu/
Swedish FBDs data	Public Health Agency of Sweden	https://www.folkhalsomyndigheten.se/folkhalsorapportering-statistik/
European FDBs data	European Centre for Disease Prevention and Control	https://atlas.ecdc.europa.eu/public/index.aspx
Poultry *Campylobacter* carriership	Swedish Veterinary Agency	https://www.sva.se/media/3xjbzi4a/smittlaget_2023_webb_v20250410.pdf

**Software and algorithms**		

SPSS 29.0.1.0	**IBM** (International Business Machines Corporation).	
Statistical analysis scripts	This paper	Statistical analyses were performed using SPSS; therefore, no custom code was generated.

### Experimental model and study participant details

#### Human surveillance data

This study used aggregated national surveillance data on six notifiable foodborne diseases in Sweden collected by the Public Health Agency of Sweden between 2013 and 2022.^11^ Available variables included age group, sex, county, and year of notification. Individual-level participant information was not available. Because only anonymized and aggregated surveillance data were analyzed, no allocation of participants to experimental groups was performed. The study population comprised all notified cases recorded within the Swedish surveillance system during the study period.

Sex-specific distributions were evaluated and are reported in the results section. Information regarding gender identity, ethnicity, ancestry, race, socioeconomic status, and individual-level clinical characteristics was not available in the surveillance datasets and therefore could not be assessed. This limitation should be considered when interpreting demographic differences and the generalizability of the findings.

#### Animal surveillance data

Data on Campylobacter carriage in broiler chickens were obtained from the Swedish Veterinary Agency national surveillance programme. The programme covers more than 90% of commercial broiler production in Sweden. Annual numbers of positive slaughter groups from 2013–2022 were analyzed in relation to human campylobacteriosis incidence. Only aggregated surveillance data were used in the analyses.

### Method details

#### Data collection

As this study aimed to examine FBDs in Sweden, we focused on a selection of six diseases: campylobacteriosis, cryptosporidiosis, EHEC infection, hepatitis A infection, listeriosis, and non-typhoidal salmonellosis. While not exhaustive, this selection includes, with some exceptions, the most reported FBDs in Sweden with foodborne transmission rates ranging from 10% to 100%.^2^

All selected diseases are notifiable under Swedish surveillance systems.^29^ Reported cases of these notifiable infections were gathered from the Public Health Agency of Sweden from 2013 to 2022.^11^ In addition, data regarding the county incidence rates per county (*n*=21), age and gender distributions were collected for all pathogens studied. Data on endemic and non-endemic cases were also collected for some FBDs.

In addition to human cases, data was collected from the Swedish Food Agency (SFA), via special request, on suspected foodborne outbreaks to identify the food types most linked to each of the pathogens studied (unpublished data from SFA). Data included suspected and confirmed foodborne cases reported by Swedish municipalities during the study period. Suspected cases where the food source contaminant was categorized as “Unknown” or “Other” were excluded. These cases were due to missing samples during inspections, difficulties in cultivation, or unidentified sources. These data reflect outbreak-based, traceable cases and should not be interpreted as population-level exposure or attribution of fractions due to the large amount of excluded data.

To explore potential links between FBDs and animals, data were collected from the Swedish Veterinary Agency (SVA). *Campylobacter* carriership in poultry is notifiable according to Swedish law and the Swedish Board of Agriculture funds a national *Campylobacter* surveillance programme. We therefore used this data for our animal data. The *Campylobacter* surveillance programme in Sweden covers over 90% of all commercial broiler chicken farms in Sweden. Annual summaries were extracted for all positive chicken slaughter groups during the study period.^30^ We did not explore surveillance programmes for other food-borne pathogens in animals, as they did not have a surveillance programme (*Cryptosporidium* and *Hepatitis A* virus) or they had a programme where monitoring was only passive (*Listeria, Salmonella*, and EHEC).^30^

Bioclimatic variables were collected to explore potential links between FBDs and climate change. Metrics were retrieved from monthly precipitation and temperature data, designed to capture biologically accurate and meaningful climate patterns. In total, 19 bioclimatic variables for Sweden were all extracted from the European Climate Data Observatory.^31^

To provide a regional overview of the studied FBDs, we extracted country-level annual notification rates (reported cases per 100,000) for Sweden, Denmark, Finland, Norway, Iceland, and the EU/EEA from the ECDC in open datasets for 2013–2022.^32^ No imputation or re-scaling was applied. Accordingly, the reported values reflect each country’s surveillance definitions, diagnostic practices, and reporting systems.

#### Quantifying FBD burden in Sweden

The burden of FBDs in Sweden was quantified using a DALYs estimation approach based on reported cases. The Underreporting Multipliers (UM), mean DALY per case and FBD attribution rate were used to estimate the total DALY for each FBD. Underreporting was adjusted by using specific UM based on Sweden-specific data.^12^ Due to the severity of symptoms in listeriosis, no UM was applied, as it was assumed that all cases were captured in the ECDC database, an assumption commonly made in other studies.^12^^,^^14^ For some FBDs that lacked specific Swedish data, UMs were extracted from previous European studies.^13^^,^^14^ DALY per case data was extracted from WHO^15^ and the number was finally corrected for foodborne attribution rates per pathogen according to Hald et al.^2^

### Quantification and statistical analysis

To investigate the gender disparities among the various FBDs and identify gender vulnerabilities and susceptibilities, the (non-parametric) Kruskal-Wallis test was performed. To explore the correlation between *Campylobacter* broiler chicken carriership and human cases, two regression analyses were conducted: one for endemic human cases and another for non-endemic human cases, against the broiler chicken cases. The resulting *R*^*2*^ and *p*-values were used to evaluate the correlation and significance between the variables.

In the climate analysis, a generalized linear model (GLM) was used to identify significant bioclimatic variables influencing FBDs. The data used for the GLM consists of yearly FBDs collected from various counties in Sweden. For each year and county, relevant bioclimatic variables were extracted to capture environmental conditions. These variables were then used in the analysis to assess patterns and relationships within the dataset. Before the GLM analysis, some bioclimatic variables were removed based on correlation analyses (coefficients exceeding 0.8) (Table S7) to reduce the multicollinearity issue. Although highly correlated bioclimatic variables were removed prior to the GLM analysis, some potential for moderate multicollinearity among the retained variables may remain. It is important to note that this residual collinearity might slightly affect the interpretation of individual variable estimates and should be taken into account when evaluating the specific effects of single climatic factors. A negative binomial log distribution was used in the model. In constructing the model, the year and county were included as factors to account for temporal and spatial variability, respectively. The year and county were treated as factors to capture non-linear and spatial effects. We adjusted for population variations across counties by using the 2023 population data for each county, which was collected from Statistics Sweden.^33^

All statistical analyses were conducted using SPSS 29.0.1.0, and all statistical tests were performed with a significance level of less than 0.05.
