# Supplementary material for: Foodborne disease burden in Sweden with a One Health perspective
Source: iScience. 2026 Aug 4;29(8):117023. doi: 10.1016/j.isci.2026.117023 (PMC13463798; doi:10.1016/j.isci.2026.117023)
Supplement: Supplementary file 1 [file mmc1.pdf]

iScience, Volume *29*

Supplemental information

Foodborne disease burden in Sweden with a One Health perspective

Ismael Halawa, Anna Omazic, and Junwen Guo

Supplementary Material

Foodborne disease burden in Sweden with a One Health Perspective

Ismael Halawa[1], Anna Omazic[2], Junwen Guo[1,3*]

[1]Department of Epidemiology and Global Health, Umeå University, 901 85 Umeå, Sweden

[2]Department of Chemistry, Environment and Feed Hygiene, Swedish Veterinary Agency (SVA), 751 89 Uppsala, Sweden

[3]Lead contact

*Correspondence: junwen.guo@umu.se

Table S1. Generalized linear model (negative binomial) results for salmonellosis incidence rate ratios (IRR, 95% CI).

SALMONELLOSIS<br>Parameter (GLM: negative binomial log)	Incidence Rate Ratios (IRR)	95% Wald Confidence Interval for IRR	
		Lower	Upper
(Intercept)	0.001	7.34E-10	1382.78
[YEAR = 2013]	1.889	0.619	5.762
[YEAR = 2014]	1.147	0.404	3.259
[YEAR = 2015]	1.05	0.274	4.019
[YEAR = 2016]	1.839	0.734	4.608
[YEAR = 2017]	1.597	0.487	5.229
[YEAR = 2018]	1.48	0.306	7.154
[YEAR = 2019]	1.581	0.712	3.51
[YEAR = 2020]	0.62	0.175	2.193
[YEAR = 2021]	2.114	0.649	6.886
[YEAR = 2022]	1	.	.
[REGION =Blekinge]	1.874	0.543	6.461
[REGION =Dalarna]	9.073	0.699	117.764
[REGION =Gävlebo]	2.934	0.422	20.387
[REGION =Gotland]	6.564	1.608	26.788
[REGION =Halland]	2.824	1.035	7.706
[REGION =Jämtlan]	21.905	0.692	693.513
[REGION =Jönköp]	1.607	0.707	3.654
[REGION =Kalmar]	2.006	0.684	5.882
[REGION =Kronober]	1.948	0.881	4.305
[REGION =Norrbott]	17.475	0.134	2271.041
[REGION =Orebro]	2.905	1.115	7.569
[REGION =Österg]	1.132	0.414	3.093
[REGION =Skåne]	1.014	0.304	3.388
[REGION =Söderma]	1.64	0.534	5.042
[REGION =Stockhol]	0.995	0.348	2.845
[REGION =Uppsala]	0.796	0.247	2.563
[REGION =Värmlan]	1.552	0.332	7.252
[REGION =Västerb]	4.999	0.096	259.674
[REGION =Västern]	4.924	0.306	79.128
[REGION =Västman]	0.853	0.293	2.482
[REGION =Västra]	1	.	.
bio1	1.25	0.574	2.72
bio3	1.09	0.972	1.222
bio4	0.998	0.989	1.008
bio5	1.128	0.874	1.456
bio8	0.972	0.939	1.006
bio9	1.009	0.969	1.051
bio12	0.996	0.992	1
bio14	0.988	0.961	1.016
bio15	0.998	0.979	1.018
bio17	1.004	0.992	1.017
bio18	1.008	1.002	1.014
bio19	1	0.994	1.006

Table S2. Generalized linear model (negative binomial) results for Campylobacteriosis incidence rate ratios (IRR, 95% CI).

**CAMPYLOBACTERIOSIS**			
**Parameter (GLM: negative binomial log)**	**Incidence Rate Ratios (IRR)**	**95% Wald Confidence Interval for IRR**	
		**Lower**	**Upper**
(Intercept)	0.025	2.60E-08	23793.37
[YEAR = 2013]	2.001	0.679	5.898
[YEAR = 2014]	1.217	0.435	3.403
[YEAR = 2015]	0.698	0.189	2.585
[YEAR = 2016]	1.645	0.683	3.963
[YEAR = 2017]	1.353	0.43	4.255
[YEAR = 2018]	2.283	0.49	10.638
[YEAR = 2019]	1.494	0.681	3.275
[YEAR = 2020]	0.467	0.133	1.644
[YEAR = 2021]	3.05	0.968	9.612
[YEAR = 2022]	1	.	.
[REGION =Blekinge]	0.907	0.27	3.045
[REGION =Dalarna]	7.509	0.616	91.47
[REGION =Gävlebo]	2.514	0.38	16.624
[REGION =Gotland]	4.854	1.215	19.398
[REGION =Halland]	2.442	0.922	6.468
[REGION =Jämtlan]	27.729	0.975	788.487
[REGION =Jönköp]	1.463	0.66	3.241
[REGION =Kalmar]	1.83	0.65	5.152
[REGION =Kronober]	1.851	0.856	4.004
[REGION =Norrbott]	34.403	0.295	4012.671
[REGION =Orebro]	2.809	1.103	7.151
[REGION =Österg]	0.864	0.323	2.309
[REGION =Skåne]	0.724	0.223	2.352
[REGION =Söderma]	1.051	0.351	3.15
[REGION =Stockhol]	0.754	0.27	2.104
[REGION =Uppsala]	0.679	0.217	2.131
[REGION =Värmlan]	1.623	0.359	7.335
[REGION =Västerb]	8.427	0.187	379.679
[REGION =Västern]	5.751	0.387	85.456
[REGION =Västman]	0.803	0.283	2.279
[REGION =Västra]	1	.	.
bio1	1.318	0.623	2.788
bio3	1.101	0.984	1.233
bio4	0.996	0.987	1.005
bio5	1.096	0.855	1.405
bio8	0.966	0.935	0.999
bio9	1	0.962	1.04
bio12	0.995	0.991	0.999
bio14	0.986	0.96	1.013
bio15	1.003	0.984	1.023
bio17	1.009	0.997	1.022
bio18	1.009	1.003	1.015
bio19	0.998	0.992	1.004

Table S3. Generalized linear model (negative binomial) results for Listeriosis incidence rate ratios (IRR, 95% CI).

**LISTERIOSIS**			
**Parameter (GLM: negative binomial log)**	**Incidence Rate Ratios (IRR)**	**95% Wald Confidence Interval for IRR**	
		**Lower**	**Upper**
(Intercept)	0.51	2.91E-07	893407.3
[YEAR = 2013]	1.133	0.375	3.422
[YEAR = 2014]	0.881	0.299	2.595
[YEAR = 2015]	0.32	0.084	1.221
[YEAR = 2016]	1.203	0.486	2.978
[YEAR = 2017]	0.658	0.206	2.103
[YEAR = 2018]	2.671	0.543	13.132
[YEAR = 2019]	1.152	0.515	2.575
[YEAR = 2020]	0.41	0.112	1.511
[YEAR = 2021]	1.626	0.504	5.243
[YEAR = 2022]	1	.	.
[REGION =Blekinge]	1.023	0.285	3.674
[REGION =Dalarna]	0.899	0.067	11.989
[REGION =Gävlebo]	0.843	0.12	5.929
[REGION =Gotland]	3.758	0.912	15.482
[REGION =Halland]	1.692	0.637	4.494
[REGION =Jämtlan]	1.496	0.047	48.023
[REGION =Jönköp]	0.54	0.232	1.258
[REGION =Kalmar]	0.629	0.219	1.804
[REGION =Kronober]	0.642	0.279	1.478
[REGION =Norrbott]	0.655	0.004	95.968
[REGION =Orebro]	1.281	0.492	3.334
[REGION =Österg]	0.447	0.16	1.253
[REGION =Skåne]	0.887	0.267	2.951
[REGION =Söderma]	1.156	0.386	3.462
[REGION =Stockhol]	0.65	0.23	1.836
[REGION =Uppsala]	0.722	0.226	2.312
[REGION =Värmlan]	1.004	0.206	4.891
[REGION =Västerb]	1.375	0.027	70.289
[REGION =Västern]	1.564	0.097	25.113
[REGION =Västman]	1.102	0.381	3.183
[REGION =Västra]	1	.	.
bio1	0.789	0.362	1.719
bio3	1.047	0.926	1.184
bio4	0.993	0.983	1.002
bio5	1.1	0.841	1.438
bio8	0.969	0.934	1.005
bio9	1.018	0.977	1.062
bio12	0.995	0.991	0.999
bio14	0.979	0.951	1.007
bio15	1.01	0.99	1.03
bio17	1.014	1.001	1.028
bio18	1.012	1.005	1.018
bio19	0.996	0.99	1.002

Table S4. Generalized linear model (negative binomial) results for Hepatitis A infection incidence rate ratios (IRR, 95% CI).

HEPATITIS A INFECTION<br>Parameter (GLM: negative binomial log)	Incidence Rate Ratios (IRR)	95% Wald Confidence Interval for IRR	
		Lower	Upper
[YEAR = 2013]	0.618	0.097	3.926
[YEAR = 2014]	2.437	0.455	13.043
[YEAR = 2015]	0.667	0.083	5.365
[YEAR = 2016]	0.773	0.179	3.34
[YEAR = 2017]	0.921	0.144	5.868
[YEAR = 2018]	2.615	0.21	32.589
[YEAR = 2019]	1.413	0.394	5.072
[YEAR = 2020]	1.021	0.127	8.236
[YEAR = 2021]	2.021	0.28	14.566
[YEAR = 2022]	1	.	.
[REGION =Blekinge]	0.588	0.084	4.106
[REGION =Dalarna]	1.309	0.022	78.033
[REGION =Gävlebo]	0.52	0.023	11.724
[REGION =Gotland]	1.181	0.132	10.538
[REGION =Halland]	3.371	0.726	15.667
[REGION =Jämtlan]	1.291	0.005	334.902
[REGION =Jönköp]	0.59	0.161	2.168
[REGION =Kalmar]	0.978	0.168	5.682
[REGION =Kronober]	0.262	0.068	1.012
[REGION =Norrbott]	0.622	0	1668.024
[REGION =Orebro]	1.257	0.286	5.519
[REGION =Österg]	0.492	0.098	2.465
[REGION =Skåne]	1.201	0.177	8.129
[REGION =Söderma]	0.482	0.081	2.872
[REGION =Stockhol]	0.218	0.042	1.142
[REGION =Uppsala]	0.38	0.061	2.383
[REGION =Värmlan]	0.496	0.041	6.022
[REGION =Västerb]	0.796	0.001	484.78
[REGION =Västern]	0.865	0.009	80.508
[REGION =Västman]	0.213	0.036	1.244
[REGION =Västra]	1	.	.
bio1	0.843	0.242	2.938
bio3	0.986	0.821	1.184
bio4	0.995	0.98	1.01
bio5	0.941	0.626	1.414
bio8	0.975	0.923	1.03
bio9	0.989	0.928	1.054
bio12	0.995	0.989	1.001
bio14	0.978	0.937	1.021
bio15	0.999	0.97	1.029
bio17	1.01	0.99	1.031
bio18	1.006	0.996	1.016
bio19	0.99	0.98	0.999

Table S5. Generalized linear model (negative binomial) results for EHEC INFECTION incidence rate ratios (IRR, 95% CI).

**EHEC INFECTION**<br>**Parameter (GLM: negative binomial log)**	**Incidence Rate Ratios (IRR)**	**95% Wald Confidence Interval for IRR**	
		**Lower**	**Upper**
(Intercept)	<.001	3.76E-17	59.259
[YEAR = 2013]	2.42	0.436	13.415
[YEAR = 2014]	1.237	0.269	5.696
[YEAR = 2015]	2.229	0.341	14.569
[YEAR = 2016]	1.356	0.35	5.249
[YEAR = 2017]	1.911	0.338	10.818
[YEAR = 2018]	0.375	0.036	3.893
[YEAR = 2019]	1.727	0.55	5.422
[YEAR = 2020]	1.391	0.234	8.251
[YEAR = 2021]	1.679	0.297	9.487
[YEAR = 2022]	1	.	.
[REGION =Blekinge]	0.269	0.046	1.558
[REGION =Dalarna]	0.944	0.025	35.013
[REGION =Gävlebo]	0.269	0.018	4.139
[REGION =Gotland]	2.987	0.411	21.706
[REGION =Halland]	0.542	0.11	2.669
[REGION =Jämtlan]	3.419	0.024	487.369
[REGION =Jönköp]	0.459	0.14	1.498
[REGION =Kalmar]	0.555	0.104	2.969
[REGION =Kronober]	0.825	0.264	2.572
[REGION =Norrbott]	0.879	0.001	815.122
[REGION =Orebro]	0.606	0.155	2.37
[REGION =Österg]	0.556	0.128	2.417
[REGION =Skåne]	0.697	0.127	3.834
[REGION =Söderma]	0.383	0.074	1.972
[REGION =Stockhol]	0.497	0.111	2.219
[REGION =Uppsala]	0.253	0.047	1.363
[REGION =Värmlan]	0.841	0.097	7.31
[REGION =Västerb]	0.501	0.002	126.96
[REGION =Västern]	2.189	0.044	108.884
[REGION =Västman]	0.324	0.066	1.58
[REGION =Västra]	1	.	.
bio1	1.356	0.452	4.064
bio3	1.172	0.99	1.388
bio4	1.007	0.994	1.021
bio5	1.107	0.752	1.629
bio8	0.981	0.932	1.033
bio9	1.019	0.958	1.083
bio12	0.996	0.99	1.001
bio14	1.012	0.973	1.053
bio15	1.015	0.986	1.045
bio17	1.012	0.993	1.032
bio18	0.999	0.99	1.007
bio19	1.005	0.996	1.015

Table S6. Generalized linear model (negative binomial) results for cryptosporidiosis incidence rate ratios (IRR, 95% CI).

**CRYPTOSPORIDIOSIS**			
**Parameter (GLM: negative binomial log)**	**Incidence Rate Ratios (IRR)**	**95% Wald Confidence Interval for IRR**	
		**Lower**	**Upper**
(Intercept)	17156.5	7.24E-06	4.07E+13
[YEAR = 2013]	0.853	0.178	4.084
[YEAR = 2014]	0.22	0.048	0.996
[YEAR = 2015]	0.137	0.018	1.066
[YEAR = 2016]	0.431	0.119	1.553
[YEAR = 2017]	0.298	0.056	1.575
[YEAR = 2018]	1.951	0.164	23.267
[YEAR = 2019]	0.573	0.174	1.879
[YEAR = 2020]	0.222	0.034	1.432
[YEAR = 2021]	0.759	0.15	3.847
[YEAR = 2022]	1	.	.
[REGION =Blekinge]	0.059	0.01	0.362
[REGION =Dalarna]	0.22	0.007	7.207
[REGION =Gävlebo]	0.082	0.005	1.368
[REGION =Gotland]	2.797	0.372	21.003
[REGION =Halland]	0.359	0.08	1.602
[REGION =Jämtlan]	0.46	0.003	64.764
[REGION =Jönköp]	0.204	0.063	0.66
[REGION =Kalmar]	1.229	0.241	6.266
[REGION =Kronober]	0.504	0.162	1.569
[REGION =Norrbott]	0.297	0	326.894
[REGION =Orebro]	0.669	0.166	2.688
[REGION =Österg]	0.829	0.188	3.646
[REGION =Skåne]	0.822	0.15	4.505
[REGION =Söderma]	1.753	0.322	9.558
[REGION =Stockhol]	0.768	0.163	3.613
[REGION =Uppsala]	0.604	0.107	3.419
[REGION =Värmlan]	0.735	0.079	6.84
[REGION =Västerb]	0.196	0.001	47.626
[REGION =Västern]	0.379	0.008	18.587
[REGION =Västman]	0.76	0.154	3.749
[REGION =Västra]	1	.	.
bio1	0.517	0.164	1.632
bio3	0.931	0.779	1.113
bio4	0.986	0.972	1
bio5	1.228	0.837	1.802
bio8	0.939	0.895	0.985
bio9	0.971	0.916	1.029
bio12	0.995	0.989	1
bio14	1.013	0.971	1.057
bio15	1.032	1.002	1.063
bio17	1.03	1.01	1.051
bio18	1.009	0.999	1.018
bio19	0.994	0.985	1.002

Table S7. Pearson correlation matrix of the 19 bioclimatic variables.

	bio1	bio2	bio3	bio4	bio5	bio6	bio7	bio8	bio9	bio10	bio11	bio12	bio13	bio14	bio15	bio16	bio17	bio18	bio19
bio1	1.00	-0.89	-0.20	-0.72	0.34	0.94	-0.82	0.01	0.67	0.85	0.94	0.08	0.03	-0.17	0.05	0.01	0.04	-0.25	0.27
bio2	-0.89	1.00	0.38	0.69	-0.12	-0.89	0.85	0.00	-0.58	-0.71	-0.85	-0.08	-0.03	0.12	-0.06	-0.05	-0.02	0.23	-0.21
bio3	-0.20	0.38	1.00	-0.34	-0.49	0.00	-0.17	-0.19	-0.10	-0.49	0.06	0.32	0.25	0.10	0.02	0.30	0.26	0.29	0.27
bio4	-0.72	0.69	-0.34	1.00	0.20	-0.86	0.93	0.15	-0.51	-0.28	-0.90	-0.38	-0.28	-0.02	-0.05	-0.30	-0.31	-0.03	-0.47
bio5	0.34	-0.12	-0.49	0.20	1.00	0.17	0.17	0.16	0.30	0.63	0.11	-0.25	-0.09	-0.07	0.09	-0.26	-0.17	-0.29	-0.04
bio6	0.94	-0.89	0.00	-0.86	0.17	1.00	-0.94	-0.06	0.66	0.68	0.97	0.19	0.14	-0.09	0.09	0.13	0.12	-0.17	0.37
bio7	-0.82	0.85	-0.17	0.93	0.17	-0.94	1.00	0.11	-0.56	-0.47	-0.93	-0.28	-0.17	0.07	-0.06	-0.22	-0.17	0.07	-0.38
bio8	0.01	0.00	-0.19	0.15	0.16	-0.06	0.11	1.00	-0.01	0.09	-0.09	-0.15	-0.02	0.04	0.09	-0.11	-0.10	0.19	-0.22
bio9	0.67	-0.58	-0.10	-0.51	0.30	0.66	-0.56	-0.01	1.00	0.58	0.66	0.12	0.10	0.00	0.00	0.05	0.13	-0.19	0.43
bio10	0.85	-0.71	-0.49	-0.28	0.63	0.68	-0.47	0.09	0.58	1.00	0.65	-0.16	-0.16	-0.31	0.04	-0.22	-0.18	-0.41	0.06
bio11	0.94	-0.85	0.06	-0.90	0.11	0.97	-0.93	-0.09	0.66	0.65	1.00	0.21	0.13	-0.10	0.03	0.12	0.18	-0.17	0.40
bio12	0.08	-0.08	0.32	-0.38	-0.25	0.19	-0.28	-0.15	0.12	-0.16	0.21	1.00	0.74	0.42	-0.09	0.90	0.71	0.72	0.74
bio13	0.03	-0.03	0.25	-0.28	-0.09	0.14	-0.17	-0.02	0.10	-0.16	0.13	0.74	1.00	0.36	0.45	0.84	0.40	0.67	0.54
bio14	-0.17	0.12	0.10	-0.02	-0.07	-0.09	0.07	0.04	0.00	-0.31	-0.10	0.42	0.36	1.00	-0.30	0.32	0.53	0.46	0.31
bio15	0.05	-0.06	0.02	-0.05	0.09	0.09	-0.06	0.09	0.00	0.04	0.03	-0.09	0.45	-0.30	1.00	0.23	-0.48	0.04	-0.18
bio16	0.01	-0.05	0.30	-0.30	-0.26	0.13	-0.22	-0.11	0.05	-0.22	0.12	0.90	0.84	0.32	0.23	1.00	0.48	0.73	0.58
bio17	0.04	-0.02	0.26	-0.31	-0.17	0.12	-0.17	-0.10	0.13	-0.18	0.18	0.71	0.40	0.53	-0.48	0.48	1.00	0.44	0.66
bio18	-0.25	0.23	0.29	-0.03	-0.29	-0.17	0.07	0.19	-0.19	-0.41	-0.17	0.72	0.67	0.46	0.04	0.73	0.44	1.00	0.38
bio19	0.27	-0.21	0.27	-0.47	-0.04	0.37	-0.38	-0.22	0.43	0.06	0.40	0.74	0.54	0.31	-0.18	0.58	0.66	0.38	1.00
